# Decoding Gen Z: AI's influence on brand trust and purchasing behavior

**DOI:** 10.3389/frai.2024.1323512

**Published:** 2024-03-04

**Authors:** Cristobal Rodolfo Guerra-Tamez, Keila Kraul Flores, Gabriela Mariah Serna-Mendiburu, David Chavelas Robles, Jorge Ibarra Cortés

**Affiliations:** ^1^Art and Design Department, Centro Roberto Garza Sada de Arte, Arquitectura y Diseño, Universidad de Monterrey, Nuevo León, Mexico; ^2^Department of Marketing and Analysis, Instituto Tecnológico y de Estudios Superiores de Monterrey, Monterrey, Nuevo León, Mexico

**Keywords:** artificial intelligence, brand trust, flow experience, consumer behavior, Gen Z

## Abstract

This study focuses on the role of AI in shaping Generation Z's consumer behaviors across fashion, technology, beauty, and education sectors. Analyzing responses from 224 participants, our findings reveal that AI exposure, attitude toward AI, and AI accuracy perception significantly enhance brand trust, which in turn positively impacts purchasing decisions. Notably, flow experience acts as a mediator between brand trust and purchasing decisions. These insights underscore the critical role of AI in developing brand trust and influencing purchasing choices among Generation Z, offering valuable implications for marketers in an increasingly digital landscape.

## 1 Introduction

In today's digital era, Artificial Intelligence (AI) is revolutionizing consumer-brand relationships, particularly among Generation Z. Born into a world of technology, this generation engages with AI in ways that profoundly transform their consumption behaviors and expectations. The integration of AI into marketing practices is a burgeoning phenomenon, critical for understanding and adapting to the evolving dynamics of consumer behavior. This research is timely and relevant as it delves into how AI is shaping these new dynamics, especially among younger, tech-savvy consumers.

The advent of Artificial Intelligence (AI) is particularly evident in its transformative shift in consumer behavior, most notably among Generation Z. Born between 1997 and 2005, this demographic segment emerges as a pivotal market force, distinguished by their innate digital fluency and substantial future spending power. Their unique expectations and sophistication in technology usage, especially regarding AI, make them a critical group for study in marketing and emerging technology contexts. This paper studies into the complex interplay between AI and Generation Z's consumer behavior. It scrutinizes how AI exposure, attitude toward AI and AI accuracy perception, along with the mediating effect of flow experience, shape brand trust. This trust, in turn, is examined for its impact on purchasing decisions. The exploration of these relationships aims to shed light on the multifaceted dynamics of AI's influence on consumer behavior, particularly in the context of brand trust and its downstream effects. Understanding Generation Z is vital for brands aiming to adapt and thrive in the digital era, as their consumption patterns and attitudes toward AI are indicative of broader market trends.

AI exposure, defined as the frequency of an individual's interaction with AI in their daily life, is a significant factor in shaping consumer behavior (Abrardi et al., 2022). Globally, AI-powered devices and services have become a ubiquitous part of consumers' lives. It is projected that the majority of customer interactions are expected to be managed without human intervention, signifying the pervasive influence of AI in our daily routines and interactions (Stephanidis, 2019).

Attitudes toward AI, which span a spectrum from fear and skepticism to enthusiasm and acceptance, have been identified as a significant determinant of consumer behavior (Mantello et al., 2023). A substantial body of research suggests that consumers globally are increasingly comfortable with AI, particularly when it enhances the convenience of their interactions with businesses (McLean et al., 2021; Meyer-Waarden and Cloarec, 2021; Pitardi and Marriott, 2021). This trend underscores the importance of understanding and addressing consumer attitudes toward AI, as these attitudes can significantly influence the acceptance and use of AI-powered services.

The perception of AI's accuracy, gauged by an individual's belief in the precision of AI's recommendations or decisions, is another critical factor influencing brand trust (Figueroa-Armijos et al., 2023). Research indicates that consumers are more likely to trust AI advice when they perceive the AI to be reliable (Ameen et al., 2021; Chi et al., 2021; Shin, 2021). This finding highlights the importance of ensuring the accuracy of AI systems, as consumer trust in these systems can significantly impact their willingness to follow AI recommendations and, ultimately, their purchasing decisions.

Flow experience, characterized by a state of complete immersion and involvement during an activity, has emerged as a relevant concept in understanding consumer interactions with AI (Nguyen et al., 2022). Research suggests that when consumers experience a state of flow while using AI-powered services, they are more likely to perceive the service as enjoyable, engaging, and valuable (Baabdullah et al., 2022; Kautish and Khare, 2022). This positive flow experience can lead to increased satisfaction, trust, and loyalty toward AI systems.

Brand trust, a key variable in this study, is considered a significant mediator in the relationship between AI and consumer behavior (Ameen et al., 2021; Hasan et al., 2021). It is measured based on the level of confidence an individual has in a brand's AI systems and the extent to which they rely on AI-based recommendations or decisions from the brand (Chi et al., 2021). Research underscores that trust in AI systems is a critical factor influencing consumers' purchasing decisions, highlighting the importance of building and maintaining brand trust in the era of AI.

The dependent variable in this study, purchasing decision, is assessed based on various factors. These include whether the individual makes a purchase (Allal-Chérif et al., 2021) and their satisfaction (Cheng and Jiang, 2020; Prentice et al., 2020) with the purchases made based on AI's recommendations. These variables are critical indicators of the effectiveness of AI systems in influencing consumer behavior and enhancing the shopping experience.

The primary objective of this research is to unravel the complex dynamics between AI and consumer behavior within Generation Z. We aim to investigate the direct effects of AI exposure, attitudes toward AI, and perceptions of AI accuracy on brand trust, as well as the consequent impact on purchasing decisions. Additionally, this study seeks to explore the role of flow experience as a mediating factor in these relationships. By achieving these objectives, the research intends to offer valuable insights into the evolving landscape of AI in marketing and provide strategic guidance for effectively engaging with the digitally adept Generation Z market.

## 2 Literature review

In the context of the theoretical framework, this section aims to provide a concise overview of the interplay between AI and Generation Z's consumer behavior. The focus of this study is to explore the relationships among key variables: AI exposure, attitude toward AI and AI accuracy perception, flow experience, brand trust and purchasing decisions. Understanding these relationships is crucial for comprehending the impact of AI on brand trust and its subsequent effects on consumer behavior. By studying into these variables, marketers can gain valuable insights to navigate the AI landscape effectively and enhance the overall consumer experience.

Generation Z's unique positioning as the first true digital natives makes their analysis imperative in studies concerning AI's impact on consumer behavior. Their integral role in shaping future market trends, coupled with their distinct consumer attitudes formed in the digital age, provides unparalleled insights into the evolving landscape of consumer-brand interactions in the AI era. This demographic's engagement with technology goes beyond mere usage; it shapes their expectations, trust, and loyalty toward brands, making their study crucial for understanding and forecasting market dynamics in the age of AI.

### 2.1 AI exposure and brand trust

Artificial Intelligence (AI) exposure refers to the frequency and extent of an individual's interaction with AI in their daily life. This exposure can occur through various channels, such as AI-powered devices, services, and applications (Abrardi et al., 2022). The ubiquity of AI in modern life has made AI exposure a significant factor in shaping consumer behavior (Rodgers et al., 2021). As AI becomes increasingly integrated into our daily routines and interactions, it is transforming the way consumers interact with brands and make purchasing decisions (McLean et al., 2021).

AI exposure is particularly relevant in the context of online business and e-commerce. For instance, in fashion and apparel industry, affiliate marketing systems, which are often AI-powered, have become a popular marketing tool (Yeo et al., 2022). These systems can increase the exposure of products and services, thereby enhancing brand visibility and credibility.

The relationship between AI exposure and brand trust has been a subject of interest in recent research (Hasan et al., 2021; Youn and Jin, 2021; Minton et al., 2022). Brand trust is a critical factor influencing consumers' purchasing decisions. In this research It will measure based on the level of confidence an individual has in a brand's AI systems and the extent to which they rely on AI-based recommendations or decisions from the brand.

The influence of AI exposure on brand trust has been substantiated by a wealth of research across various industries. In the hospitality sector, for instance, studies have found that the trust in AI-powered affiliate marketing systems can significantly impact consumers' intentions to book accommodations. This trust is often intertwined with factors such as social contact and consumers' self-efficacy in navigating the AI interface (Bhushan, 2021; Khaliq et al., 2022; Rasheed et al., 2023).

Similarly, in the realm of e-commerce, research has underscored the role of trust in online vendors and merchants in mitigating the perceived risks of online transactions (Kim et al., 2021). This trust is often shaped by consumers' perception of the benefits of AI, such as enhanced security and the reputation of the website, as well as their familiarity with the AI system (Micu et al., 2021; Fedorko et al., 2022; Fonseka et al., 2022).

In the gaming industry, where AI is increasingly used to enhance user experience, studies have shown that players' trust in the game's AI system can significantly influence their engagement with the game. This trust is often linked to the perceived fairness and competence of the AI system (Yang and Nazir, 2022; Khatri, 2023; Xia, 2023).

These findings collectively suggest that increased AI exposure can bolster brand trust, thereby influencing consumers' purchasing decisions. However, it is crucial to note that the impact of AI exposure on brand trust is multifaceted and may be contingent on other factors. These include the perceived accuracy and reliability of the AI system, the individual's attitudes toward AI, and their past experiences with AI.

In the context of Generation Z, AI exposure takes on a unique dimension, given their innate digital fluency and constant interaction with emerging technologies. This demographic, accustomed to the omnipresent AI in their daily lives, provides fertile ground to explore how this exposure affects their trust in brands. While previous studies have examined the relationship between AI exposure and brand trust, there is a significant gap in the literature regarding how this dynamic specifically unfolds within Generation Z.

Our study addresses this gap by exploring whether increased AI exposure among Generation Z consumers leads to greater brand trust. This approach not only contributes to understanding Generation Z's interaction with AI but also provides valuable insights for brand management strategies in the digital age. Therefore, we propose the following hypothesis:

Hypothesis 1: Increased AI exposure positively influences brand trust among Generation Z consumers.

### 2.2 Attitude toward AI and brand trust

Research has shown that attitudes toward AI can significantly influence brand trust (Qin et al., 2020; Ahn et al., 2022; Yang and Wibowo, 2022). For instance, in the realm of e-commerce, studies have underscored the role of trust in online vendors and merchants in mitigating the perceived risks of online transactions. This trust is often shaped by consumers' perception of the benefits of AI, such as enhanced security and the reputation of the website, as well as their familiarity with the AI system (Khrais, 2020; Nagy and Hajd, 2021; Rashidin et al., 2021).

In the context of viral marketing, the appeal and credibility of the message source, which often communicates the use of AI, have been found to significantly impact consumers' attitudes toward the brand. This suggests that the way AI is utilized and communicated in marketing messages can influence consumers' attitudes toward AI and, consequently, their trust in the brand (Hayes et al., 2021; Vlačić et al., 2021; Ameen et al., 2022).

In the airline industry, studies have shown that customers' attitudes toward traditional and social media marketing, where the use of AI is often communicated, can affect brand trust and purchase intention. This indicates that the medium through which AI is presented and its use is communicated can influence consumers' attitudes toward AI and their trust in the brand (Rana et al., 2021; Singh, 2021).

These findings collectively suggest that attitudes toward AI can significantly influence brand trust. However, it is crucial to note that the impact of attitudes toward AI on brand trust is multifaceted and may be contingent on other factors. These include the perceived accuracy and reliability of the AI system, the individual's past experiences with AI, and their exposure to AI. As such, a comprehensive understanding of these dynamics is essential for leveraging AI effectively to build brand trust and enhance consumer satisfaction.

In the domain of Generation Z, attitudes toward AI play a pivotal role in shaping brand trust. This demographic, known for its adaptability to technology, may demonstrate unique perspectives on AI, influencing their trust in brands utilizing AI technologies. Studies in e-commerce and viral marketing have highlighted how consumers' perceptions of AI, including its benefits and application, shape their trust in online vendors and brand messages (McLean et al., 2021; Marjerison et al., 2022). Additionally, in industries like airlines, the way AI is communicated through marketing channels has a significant impact on brand trust and purchase intentions (Tussyadiah and Miller, 2019).

These findings suggest that Generation Z's attitudes toward AI, influenced by the factors such as AI's perceived accuracy and past experiences, are crucial in building brand trust. This study extends this understanding by examining how positive attitudes toward AI among Generation Z consumers can enhance brand trust. Therefore, we propose the following hypothesis:

Hypothesis 2: Positive attitudes toward AI positively influence brand trust among Generation Z consumers.

### 2.3 AI accuracy perception and brand trust

Artificial Intelligence (AI) accuracy perception refers to an individual's belief in the precision of AI's recommendations or decisions (Nadarzynski et al., 2019). This perception is a critical factor in shaping consumer behavior and influencing brand trust (Pelau et al., 2021). As AI systems become increasingly sophisticated and accurate, they are transforming the way consumers interact with brands and make purchasing decisions (Wang et al., 2022).

AI accuracy perception is particularly relevant in the context of online business and e-commerce. For instance, in the airline industry, studies have shown that customers' attitudes toward AI, as presented through traditional and social media marketing, can affect brand trust and purchase intention. This indicates that the medium through which AI is presented and communicated to consumers can influence their perceptions of AI's accuracy and their trust in the brand (Mayer et al., 2020; Kim et al., 2021; Strich et al., 2021).

The influence of AI accuracy perception on brand trust has been substantiated by a wealth of research across various industries. In the realm of recommender systems, studies have found that consumers are more likely to trust AI advice when they perceive the AI to be reliable (Abbass, 2019; Shi et al., 2021). This trust is often intertwined with factors such as the perceived benefits of AI, such as enhanced security and the reputation of the website, as well as their familiarity with the AI system (Cabiddu et al., 2022).

In the context of social media influencers, research has shown that consumers' attitudes toward AI influencers can significantly impact brand trust and purchase intention (Alboqami, 2023). AI accuracy perception, gauged by an individual's belief in the precision of AI's recommendations or decisions, is pivotal in shaping brand trust (Kim et al., 2021). In marketing communications, the manner in which AI is incorporated can sway consumers' perceptions of its accuracy (Cheng and Jiang, 2022). If AI is used to provide personalized, accurate product recommendations, it could enhance consumers' perception of the brand's AI accuracy, thereby boosting their trust in the brand. This trust can subsequently influence purchasing decisions (Kumar et al., 2019).

These findings collectively suggest that positive perceptions of AI's accuracy can bolster brand trust, thereby influencing consumers' purchasing decisions. However, it is crucial to note that the impact of AI accuracy perception on brand trust is multifaceted and may be contingent on other factors. These include the individual's attitudes toward AI, their past experiences with AI, and their exposure to AI. As such, a comprehensive understanding of these dynamics is essential for leveraging AI effectively to build brand trust and enhance consumer satisfaction.

In the digital landscape where Generation Z is a significant player, the perception of AI's accuracy is crucial in shaping brand trust. This demographic, known for its critical engagement with technology, values the precision of AI systems in their interactions with brands (Guo and Luo, 2023). Research across various sectors, including e-commerce and retail, highlights that when AI is perceived as accurate and reliable, it significantly enhances consumers' trust in brands (Ho and Chow, 2023; Nazir et al., 2023).

For Generation Z, the way AI is presented and its perceived reliability in providing personalized and accurate recommendations are key factors influencing their trust in a brand. This study extends these findings by examining the impact of AI accuracy perception on brand trust among Generation Z consumers, offering insights into how their unique perceptions of AI shape brand relationships. Therefore, we propose the following hypothesis:

Hypothesis 3: Positive AI accuracy perception positively influences brand trust among Generation Z consumers.

### 2.4 Brand trust and purchasing decisions

Brand trust encapsulates the faith and confidence consumers harbor regarding the sincerity and integrity embodied in a brand's actions and communications (Portal et al., 2019). This trust extends to an anticipation that the brand will act favorably, even in scenarios where consumers lack control or face uncertainty regarding the outcomes (Gretry et al., 2017).

The pivotal role of brand trust lies in its capacity to foster and sustain long-term relationships with consumers (Menidjel et al., 2017). Trust in a brand ameliorates perceived risk associated with purchasing decisions (Kim and Chao, 2019; Arruda Filho et al., 2020), thereby cultivating a more loyal consumer base. This loyalty often translates to repeat purchases and favorable recommendations (Quaye et al., 2022). Moreover, consumers exhibit a willingness to pay a premium for products from trusted brands, attributing additional value to the safety and quality these brands epitomize (Chakraborty, 2019).

The nexus between brand trust and purchasing decisions has garnered substantial attention in marketing academia, particularly within the e-commerce milieu. Empirical evidence posits that brand trust exerts a direct influence on consumers' purchase intentions (Zuech et al., 2015; Zhao et al., 2019; Nosi et al., 2021). For instance, a notable study delineated the mediating role of brand trust in the relationship between perceived website quality and purchase intention (Qalati et al., 2021).

Similarly, investigations within the retail sector have unveiled a significant impact of brand trust on consumer loyalty, which subsequently shapes repeat purchase behaviors (Diallo et al., 2021). This impact is particularly pronounced in high-risk product categories, where consumers' reliance on a brand's reliability and consistency is paramount (Dabholkar and Sheng, 2012).

Moreover, the extant literature posits that brand trust can serve as a bulwark against adverse information. In instances of negative publicity or unfavorable product reviews, consumers with established trust in the brand are more inclined to adopt a lenient stance or interpret the adversities less harshly (Folse et al., 2013; Bhandari and Rodgers, 2020).

In the context of Generation Z, brand trust becomes increasingly significant. Known for their discerning nature and reliance on digital information, Gen Z's trust in a brand heavily influences their purchasing decisions (Serravalle et al., 2022; Pradhan et al., 2023). This demographic, more than previous generations, values authenticity and integrity in brand communications, which in turn shapes their buying behavior. Trust alleviates perceived risks, fostering loyalty and a propensity for repeat purchases among Gen Z consumers (Ismail et al., 2021; Joshi and Garg, 2021).

Empirical studies within the digital marketplace have shown a direct correlation between brand trust and purchase intentions for Gen Z, particularly in online environments where trust is paramount (Tabassum et al., 2020; Kim-Vick and Yu, 2023). Our study aims to further investigate this relationship, proposing the following hypothesis:

Hypothesis 4: High brand trust positively influences purchasing decisions of Generation Z consumers.

### 2.5 Flow experience as a mediating variable in brand trust and the purchasing decisions

Flow experience, a concept introduced by Csikszentmihalyi and Csikszentmihalyi (2014), refers to a state of complete immersion and involvement during an activity, characterized by a loss of self-consciousness and a sense of optimal experience. In the context of AI interactions, flow experience can be understood as the degree to which an individual is fully engaged and absorbed in the interaction with the AI system (Baabdullah et al., 2022). This experience is often associated with a sense of enjoyment, engagement, and satisfaction (Kautish and Khare, 2022).

The importance of flow experience in AI interactions lies in its potential to enhance the user's perception of the AI system and their overall experience (Ashfaq et al., 2020). When users experience a state of flow during their interaction with an AI system, they are more likely to perceive the system as enjoyable, engaging, and valuable (Nguyen et al., 2022). This positive flow experience can lead to increased satisfaction, trust, and loyalty toward the AI system, thereby influencing their future interactions and decisions related to the AI system (Sampat et al., 2023).

The relationship between flow experience and brand trust has been explored in various retail and e-commerce contexts. Research has indicated that consumers' flow experience can significantly impact their trust in brands (Shim et al., 2015; Guerra-Tamez and Franco-García, 2022). This trust is often shaped by consumers' perception of the brand's benefits, such as its reputation, quality of products or services, and their familiarity with the brand (Iglesias et al., 2019).

Empirical research has shown that when consumers perceive a brand as useful and relevant, they are more likely to experience a state of flow. This flow experience, in turn, has a substantial positive impact on their brand loyalty. For example, in Bilgihan (2016) it states that consumers' flow experience significantly influenced their trust in a brand, which subsequently affected their loyalty to the brand (Bilgihan et al., 2014). Other research by Ozkara et al. (2017) indicated that consumers' flow experience during their interaction with a brand positively influenced their trust in it, which then influenced their purchase intention. These studies underscore that fostering a positive flow experience for consumers can critically enhance brand trust and loyalty. Likewise, it has also been proven that the positive flow experience has been indicated as a mediating variable in this relationship, which suggests that a positive flow experience can increase trust in the brand and, consequently, affect repurchase intentions (Bilgihan, 2016).

**Figure 1:**
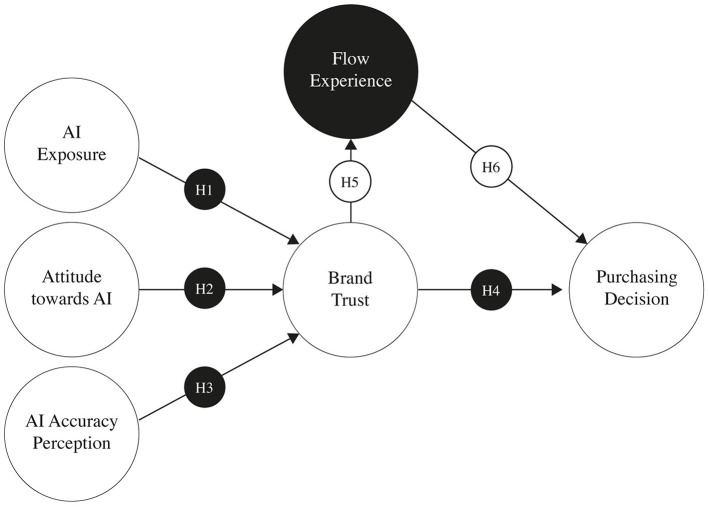
Conceptual framework.

Flow experience, defined as a state of complete immersion in an activity, holds particular significance in the context of Generation Z's interactions with AI systems. This research explores how flow experience may mediate the relationship between brand trust and purchasing decisions among this demographic. With Generation Z's inherent familiarity with digital technology, their flow experience in AI interactions could uniquely influence their brand trust and purchasing behaviors. While flow experience has been studied in various contexts, its specific application to Generation Z's AI interactions is a less explored area. Therefore, we propose:

Hypothesis 5: Positive flow experiences mediate the relationship between brand trust and purchasing decisions among Generation Z consumers. The relationships formulated in this study are shown in Figure 1.

## 3 Methodology

### 3.1 Data collection

The choice to focus on the Generation Z demographic in this study is rooted in their unique characteristics as digital natives and their emerging influence in the consumer market. This generation's deep integration with digital technology, particularly AI, offers a distinct perspective on brand trust and purchasing behaviors. Their interactions with AI in online shopping provide a rich context for examining the nuances of AI's impact on consumer behavior (Ameen et al., 2023). The insights gained from this focus are instrumental in understanding the evolving landscape of AI in marketing and consumer engagement.

To initiate the data collection process, preliminary outreach was made to several prominent universities in Mexico. Formal invitation letters were dispatched to the academic and alumni relations departments of these institutions, seeking their collaboration and consent for data collection. Out of the institutions approached, Universidad de Monterrey, Universidad Autónoma de Nuevo León and Tecnológico de Monterrey granted permission to proceed with the research.

To ensure the specificity of our study's focus on Generation Z, stringent selection criteria were employed. The target respondents were defined as individuals belonging to Generation Z, specifically those aged between 18 and 26 during the data collection period (July-September 2023), aligning with the birth years 1997 to 2005. This age criterion was a key determinant in the participant selection process, and initial filtering questions were included at the beginning of the survey to confirm the respondent's age group. A post-survey data verification process was also implemented to further validate that participants fell within the Generation Z age bracket, maintaining the integrity of our targeted demographic study.

The survey instrument was initially drafted in English and then translated into Spanish to cater to the primary language of the target audience. To ensure the accuracy and relevance of the survey items, a back translation method was employed.

To mitigate the potential for common method bias in our study, we carefully designed our survey to include procedural remedies. This involved randomizing the order of the questions to reduce patterned responses. Additionally, we used varied question formats to prevent response biases that can arise from monotonous answering patterns. These measures were implemented to ensure that our data accurately reflects the independent contributions of each variable, enhancing the validity of our findings.

Further refinement of the questionnaire was achieved through in-depth interviews with both academic representatives and a select group of Generation Z students and alumni. A pilot study, involving 40 participants, was also executed to ascertain face validity and the clarity of the questionnaire's wording.

Given the digital nature of the platforms and the tech-savviness of the Generation Z demographic, an online survey was chosen as the mode of data collection. The survey was distributed via university email systems and alumni networks, with the cooperation of the participating institutions. After a span of 3 months (July-September) dedicated to data collection, a total of 224 filled-out questionnaires were retrieved, marking them ready for data analysis. This sample size was deemed adequate, considering the multiple variables in the proposed model and the recommendations from previous literature on sample sizes for comprehensive data analysis. Table 1 shows the technical information of the study.

**Table 1:** Technical information.

**Scope**	**Gen Z**
Universe	Mexican Gen Z digital consumers
Method	Questionnarie Survey
Sample size	224 valid surveys
Data field work	July-September 2023
Statistics	Collinearity statistics, CFA, PLS—SEM and invariance of measurement instrument.
Measures (7 points likert)	Purchasing decision (Zhao et al., 2019); Brand trust (Cheng and Jiang, 2022); Flow experience (Guerra-Tamez et al., 2020; Guerra-Tamez and Franco-García, 2022); AI Exposure (Kim et al., 2021); AI Accuracy Perception (Cheng and Jiang, 2022); Attitude toward (Youn and Jin, 2021).
Statistics software	Smart PLS 4.0 and SPSS Statistics 29

### 3.2 Measurement

In our study, we operationalized several constructs, as delineated in Table 2. These constructs encompass AI exposure, attitude toward AI, AI accuracy perception, brand trust, flow experience, and purchasing decision. Each of these was gauged using a seven-point Likert scale, anchored at 1 (strongly disagree) and culminating at 7 (strongly agree).

**Table 2:** Scale items.

**Constructs**	**Label**	**Scale items**
Purchasing decision (Zhao et al., 2019)	PD1	I purchase based on AI recommendations.
	PD2	AI influences my buying decisions.
	PD3	I trust my AI-aided purchasing decisions.
	PD4	AI-based purchase recommendations satisfy me.
Brand trust (Cheng and Jiang, 2022)	BT1	I trust brands using AI technology.
	BT2	Brands using AI offer reliable products/services.
	BT3	I trust product recommendations from AI-powered brands.
	BT4	Knowing a brand uses AI reassures me.
Flow experience (Guerra-Tamez et al., 2020; Guerra-Tamez and Franco-García, 2022)	FE1	I'm not distracted on AI-powered websites/apps while shopping.
	FE2	I enjoy AI-powered online shopping interactions.
	FE3	I lose track of time on AI-powered shopping sites/apps.
	FE4	I feel in control on AI-powered shopping platforms.
AI accuracy perception (Cheng and Jiang, 2022)	AAP1	AI's product recommendations are accurate.
	AAP2	AI's product suggestions are highly appropriate for me.
	AAP3	AI's information aligns with my preferences.
	AAP4	AI understands my shopping needs and preferences.
AI exposure (Kim et al., 2021)	AE1	I often interact with AI-powered devices or services.
	AE2	AI is a central part of my daily life.
	AE3	I frequently use AI for shopping.
	AE4	I am familiar with AI technology in my daily life.
Attitude toward AI (Youn and Jin, 2021)	ATA1	AI enhances my shopping experience.
	ATA2	I'm comfortable interacting with AI during shopping.
	ATA3	I trust AI-driven product suggestions.
	ATA4	AI accurately provides product recommendations.

All constructs were tested using 4 items. The dimension of AI exposure was probed drawing inspiration from a framework proposed by a specific author. The construct of attitude toward AI was shaped leveraging measures conceived by another distinguished author. The metrics to discern AI accuracy perception were tailored based on the groundwork laid by yet another author. We turned to established literature, referencing a specific author, to delineate the measures for brand trust. The items gauging flow experience found their genesis in the works of a particular author. Concluding our set of constructs, the purchasing decision was assessed using metrics aligned with the insights of a certain author.

Our survey instrument was initially crafted in English. To ensure cultural relevance and accuracy, it was subsequently translated into Spanish by a native Mexican academic. A second native Mexican scholar, well-versed in English and immersed in an English-speaking milieu, further reviewed, and refined the translated version. To bolster the robustness of our data collection we employed strategies such as randomizing the sequence of certain items and introducing variations in the phrasing within the primary survey instrument.

## 4 Data analysis and results

### 4.1 Sample profile

A comprehensive survey was conducted, gathering responses from a diverse group of 224 participants. The gender distribution revealed that the majority, 67.1%, were females, followed by 27.3% males and a smaller segment of 5.6% identifying as other. Age-wise, the largest group, 56.6%, fell into the 18–20 age bracket, with those under 18 years accounting for 13.3%. The majority had completed high school, comprising 70.6% of the sample, while 28% had obtained a college degree, and a mere 1.4% held a master's degree. When examining occupation, a striking 88.8% were students, with minimal representation from full-time and part-time workers at 2.8% and 7% respectively. On the topic of online activity, 36.4% spent 3–4 h daily, closely followed by 35.7% investing 5–6 h. Most participants displayed a medium level of tech-savviness (63.6%), with 34.3% considering themselves highly familiar with technology. In terms of online purchasing behavior, 43.4% had been shopping online for over 3 years. The primary online purchases were in fashion, accounting for 63.6% of the sample, followed by tech and mobile apps at 17.5% and beauty products at 11.9% (Full results are shown in Table 3).

**Table 3:** Sample profile (*N* = 224).

		**Frequency**	**Valid percentage**
Gender	Female	164	67.1%
	Male	66	27.3%
	Other	14	5.6%
Age	< 18	32	13.3%
	18–20	138	56.6
	21–23	61	25.2%
	24–26	7	2.8%
	> 26	5	2.1%
Education	High school graduate	173	70.6%
	College graduate	68	28,00%
	Master graduate	3	1.4%
Occupation	Full-time	7	2.8%
	Part-time	17	7,00%
	Unemployed	3	1.4%
	Student	217	88.8%
Time online at day	< 1 h	3	1.4%
	1–2 h	38	15.4%
	3–4 h	89	36.4%
	5–6 h	87	35.7%
	> 7 h	27	11.2%
Level of familiarity with technology	Low	5	2.1%
	Medium	155	63.6%
	High	84	34.3%
Time form first online purchase	< 1 Year	49	20.2%
	1–2 Years	43	17.5%
	2–3 Years	46	18.9%
	> 3 Years	106	43.4%
Type of your purchases online	Fashion	155	63.6%
	Technology and Mobile APPs	43	17.5%
	Beauty	29	11.9%
	Books and education products	17	7%

### 4.2 Measurement model

The suggested framework was corroborated using a CFA on the entire dataset through the PLS Algorithm in Smart PLS4.0 and SPSS Statistics 29. The key findings from this analysis, alongside the descriptive metrics for the constructs examined in the framework, are highlighted in Table 4. The standardized coefficients (β) exceeded 0.715, marking an optimal situation. On employing both Smart PLS 4.0 and SPSS Statistics 29, the Cronbach alpha values ranged between 0.713 and 0.827. These figures are deemed satisfactory as per existing literature. The constructs' composite dependability surpassed 0.822, and the average variance extracted (AVE) for each construct exceeded 0.537. Hence, the robustness of the constructs in our study's framework for the entire dataset stands affirmed. Moreover, the model's fit is in line with expectations, registering above 0.90 for the NFI metric at 0.931 and below 0.08 for the SRMR at 0.071.

**Table 4:** Loadings, Cronbach's Alpha, composite reliability, and AVE values.

**Construct**	**Item**	**Loadings**	**Cronbhach's alpha**	**rho_A**	**Composite reliability**	**AVE**
Purchasing decision	PD 1	0.782	0.803	0.820	0.870	0.626
	PD 2	0.749				
	PD 3	0.826				
	PD 4	0.805				
Brand trust	BT1	0.827	0.792	0.796	0.865	0.617
	BT2	0.760				
	BT3	0.745				
	BT4	0.806				
Flow experience	FE1	0.769	0.760	0.762	0.847	0.581
	FE2	0.751				
	FE3	0.774				
	FE4	0.755				
AI accuracy perception	AAP 1	0.715	0.739	0.750	0.836	0.576
	AAP 2	0.727				
	AAP 3	0.745				
	AAP 4	0.805				
AI exposure	AE1	0.781	0.722	0.730	0.827	0.545
	AE2	0.764				
	AE3	0.733				
	AE4	0.715				
Attitude toward AI	ATA1	0.714	0.713	0.722	0.822	0.537
	ATA2	0.750				
	ATA3	0.723				
	ATA4	0.789				

### 4.3 Structural equation modeling

**Figure 2:**
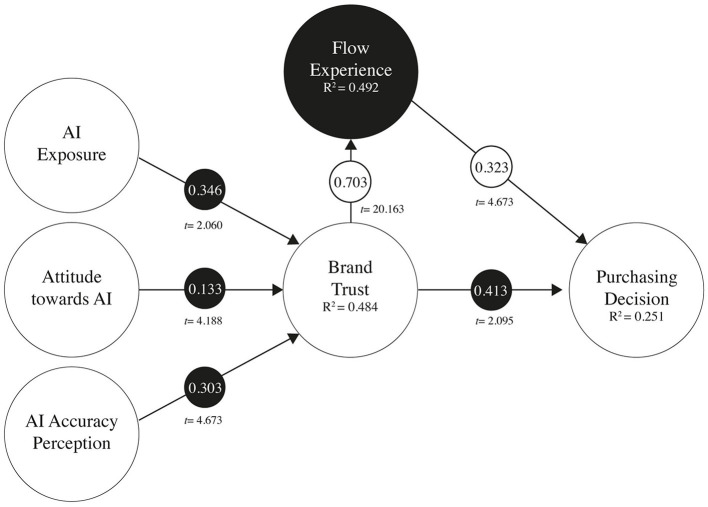
Structural model (Smart PLS 4.0). Model estimates of structural equations *p* < 0.01, Standardized Coefficient (*t*-value), continuous line: significant trajectory.

Following CFA, the structural model was tested. The hypothesized relationships in the research model have been contrasted using bootstrapping analysis via the Smart PLS 4.0 software. The results for the sample are presented in Table 5 and Figure 2, and according to the SEM analysis, all the relationships proposed in the research model have been contrasted successfully.

**Table 5:** SEM results.

**H**	**Description**	**β**	***t* Value**	***p-*Value**	**Decision**
H1	Ai exposure → brand trust	0.346	2.060	0.030	Supported
H2	Attitude toward AI → brand trust	0.133	4.188	0.000	Supported
H3	Ai accuracy perception → brand trust	0.303	4.673	0.000	Supported
H4	Brand trust → purchasing decision	0.703	2.095	0.000	Supported
H5	Brand trust → flow experience	0.413	20.163	0.000	Supported
H6	Flow experience → purchasing decision	0.323	4.673	0.039	Supported
	**Constructs**	**R** ^**2**^			
	Purchasing decision	0.252			
	Brand trust	0.494			
	Flow experience	0.484			

To evaluate the intermediary role of the flow experience, we assessed its indirect influence. This is calculated as the disparity between the total effect and the direct effect, as articulated by Christian Nitzl (Nitzl et al., 2016). As depicted in Table 6, the outcomes were significant. Given the significant outcomes in the direct effect of the brand trust and purchasing decision, the flow experience is confirmed as a partial mediating variable, thereby supporting hypotheses H6.

**Table 6:** Mediating effect of flow experience.

**H**	**Mediator**	**Independent variable**	**Dependent variable**	**β**	**t value**	***p*-value**	**Type of mediation**
H6	Flow experience	Brand Trust	Purchasing Decision	0.145	3.282	0.001	Partial mediation

### 4.4 Validation of the measuring instrument

The discriminant validity was assessed based on the criteria set by Fornell and Larcker. In the diagonal, the AVE values were placed to evaluate them against other factors in the correlation coefficient. Findings revealed values exceeding 0.5, validating the discriminant nature of all the factors (see Table 7).

**Table 7:** Discriminant validity—Fornell and Larcker criterion.

	**AE**	**APP**	**ATA**	**BT**	**FE**	**PD**
AE	**0.724**					
APP	0.645	**0.748**				
ATA	0.677	0.702	**0.734**			
BT	0.561	0.644	0.636	**0.785**		
FE	0.391	0.477	0.452	0.704	**0.762**	
PD	0.391	0.498	0.474	0.500	0.415	**0.791**

The diagonal elements, highlighted in bold, represent the square roots of the Average Variance Extracted (AVE), which indicate the amount of variance captured by a construct in relation to the variance due to measurement error. The off-diagonal elements are the inter-construct correlations. According to Fornell and Larcker's criterion for establishing discriminant validity, the square root of the AVE for each construct should exceed the correlations between that construct and any other in the model.

The collinearity statistics, as denoted by the VIF, were examined, indicating an absence of complications in the partial least squares estimations (Table 8).

**Table 8:** VIF values structural model.

	**Brand trust**	**Flow experience**	**Purchasing decision**
AI exposure	2.062		
AI accuracy perception	2.207		
Attitude toward AI	2.375		
Brand trust		1.010	1.977
Flow experience			1.977

### 4.5 Goodness-of-fit diagnosis

Tenenhaus et al. (2004) have proposed a methodology for determining the global goodness of fit (GoF). The GoF metric evaluates the congruence between a model and an observed dataset. This calculation juxtaposes observed values with those projected by the model. The method encapsulates the integrity of both the measurement and structural models (Tenenhaus et al., 2005).


$$\begin{array}{l} GoF=\sqrt[{2}]{\bar{AVE}*\bar{R^{2}}} \end{array}$$

The derived global goodness of fit (GoF) value was 0.49, surpassing the benchmark GoF of >0.36 as proposed by Wetzels et al. (2009). Consequently, it can be inferred from this investigation that the research model exhibits a satisfactory overall fit.

## 5 Discussion

In this research, we examined the influence of Artificial Intelligence (AI) interaction factors—specifically, AI exposure, attitude toward AI, and AI accuracy perception—on brand trust among Generation Z consumers across five sectors: fashion, technology and mobile apps, beauty, and books and education products. The study's methodology deployed a bootstrapping analysis to meticulously dissect the various relationships among these factors. These findings resonate with previous studies in the field, as depicted in Table 9.

**Table 9:** Authors who support the brand trust through AI.

**Constructs**	**Authors Support**
AI exposure → Brand trust	Hasan et al., 2021; Youn and Jin, 2021; Minton et al., 2022
Attitude toward AI → Brand trust	Khrais, 2020; Qin et al., 2020; Hayes et al., 2021; Nagy and Hajd, 2021; Rashidin et al., 2021; Vlačić et al., 2021; Ahn et al., 2022; Ameen et al., 2022; Yang and Wibowo, 2022
AI Accuracy Perception → Brand trust	Abbass, 2019; Shi et al., 2021; Cabiddu et al., 2022; Alboqami, 2023

Once the independent variable of brand trust was explained, its positive relationship with the dependent variable purchasing decisions that explained the university student's perception of learning was verified. Likewise, the relationship between brand trust and the flow experience was verified. These results coincide with other works in the literature, as shown in Table 10.

**Table 10:** Authors who support the purchasing decision through brand trust.

**Constructs**	**Authors support**
Brand trust → Purchasing decision	Zuech et al., 2015; Zhao et al., 2019; Nosi et al., 2021; Qalati et al., 2021

Finally, this study also verified the mediating effect of the flow experience variable between the relationships of purchasing decision. These results coincide with other works in the literature shown in Table 11.

**Table 11:** Authors who support the moderating effect of the flow experience between brand trust and purchasing decision.

**Constructs**	**Authors support**
Brand trust → Flow experience → Purchasing decision	Bilgihan et al., 2014; Shim et al., 2015; Bilgihan, 2016; Ozkara et al., 2017; Iglesias et al., 2019; Guerra-Tamez and Franco-García, 2022

Furthermore, our findings on AI's influence in the marketing sector are reflective of the broader role of AI as a disruptive technology across various industries. As detailed in the systematic literature review on AI as a disruptive technology (Păvăloaia and Necula, 2023), AI's transformative impact extends beyond marketing into sectors like healthcare, education, and urban development. This broader perspective of AI's role underscores its potential to reshape consumer interactions and expectations, particularly among digitally native populations like Generation Z. The insights gained from this study, therefore, not only contribute to understanding AI's influence in marketing but also echo AI's expansive and transformative capacity in various sectors.

### 5.1 Findings and contributions, ethical considerations, limitations, and future research suggestions

This study embarked on an exploration into the role of AI interaction factors through AI exposure, attitude toward AI, and AI accuracy perception in brand trust. Further, it delved into understanding the subsequent ripple effect this trust has on the purchasing decisions of Generation Z consumers across five distinct sectors: fashion, technology and mobile apps, beauty, and books and education products. Rooted in the belief that positive AI interactions amplify brand trust, thus swaying purchasing decisions favorably, this research uniquely positions itself at the confluence of AI and Generation Z's purchasing behavior. Through this lens, the study uncovers the transformative potential of AI on market dynamics and its broader implications for crafting marketing strategies tailored to this tech-savvy generation.

### 5.2 Findings and contributions

The research unearthed pivotal insights, establishing that AI exposure, attitude toward AI, and AI accuracy perception play a significant role in fostering brand trust. Furthermore, brand trust was found to be a robust predictor of purchasing decisions. A notable discovery was the partial mediating effect of the 'flow experience' between brand trust and purchasing decisions, adding a nuanced layer of understanding to the AI-consumer interaction dynamics.

The findings of this study are particularly illuminating when considering the unique characteristics of Generation Z. As digital natives, their interactions with AI are more intuitive and frequent compared to older generations. This inherent comfort with technology makes their responses to AI exposure, attitudes toward AI, and perceptions of AI accuracy especially relevant for marketers. Our study reveals that Generation Z's trust in brands is significantly influenced by these AI interaction factors, suggesting that marketers targeting this demographic should prioritize AI integration and transparency to build and maintain brand trust.

Moreover, the distinct purchasing behavior of Generation Z, influenced by AI, underscores the need for brands to adapt their strategies to this generation's preferences. This study's insights into the mediating role of flow experience between brand trust and purchasing decisions are particularly valuable. They suggest that creating engaging and immersive AI experiences can be a key strategy in appealing to Generation Z consumers.

These findings also open new avenues for future research. While this study focused on Generation Z, it would be insightful to compare these findings with other generational cohorts to understand generational differences in AI interaction and its impact on consumer behavior. Additionally, the unique characteristics of Generation Z identified in this study provide a valuable framework for developing targeted marketing strategies that resonate with this technologically adept generation.

In light of our study's findings, it is imperative to acknowledge the potential for alternative explanations in interpreting the influence of AI interaction factors on brand trust and purchasing decisions among Generation Z. Factors such as cultural nuances (Priporas et al., 2017), socio-economic status (Puiu et al., 2021) and individual differences in technology acceptance and digital literacy (Verma et al., 2021) may also play a moderating role in this relationship. Additionally, external market dynamics, including competitive pressures (Varsha et al., 2021) and market saturation (Guo and Luo, 2023) within the sectors analyzed, could have significant implications on consumer behavior. These considerations suggest that the interplay between AI and consumer behavior is subject to a complex matrix of variables, both internal and external to the individual.

### 5.3 Ethical implications of AI in marketing to generation Z

Alongside our findings on AI's influence on Generation Z's purchasing behavior, it's crucial to consider the ethical implications of AI in marketing. Key ethical aspects such as autonomy, the right to explanation, and value alignment, as discussed in Bertoncini and Serafim (2023), are paramount in AI systems. Generation Z, as digital natives, have heightened expectations for transparency and ethical conduct in AI interactions. Marketers should prioritize these ethical considerations to maintain trust and align with the values of this generation. The development and implementation of AI in marketing strategies should be guided by ethical principles that respect consumer autonomy, provide clear explanations of AI decisions, and align with societal values, ensuring responsible engagement with Generation Z consumers.

### 5.4 Limitations

This research, while offering crucial insights into the AI-related purchasing behaviors of Generation Z, inherently bears the limitation of focusing solely on this demographic group. While the study succeeds in providing a thorough understanding of Generation Z's unique relationship with AI, this singular focus potentially limits the generalizability of the findings across different generational cohorts. Generations like Millennials, Generation X, and Baby Boomers have their own distinct experiences and levels of technological engagement, which might lead to varying perceptions and interactions with AI. Therefore, the results of this study, though profound in the context of Generation Z, need cautious extrapolation when considering broader generational implications.

Furthermore, the adoption of a cross-sectional design was purposeful. This approach efficiently captures the current state of AI interactions, offering a clear snapshot of the present landscape. While it provides a robust overview of the current scenario, it also lays the foundation for future longitudinal studies to track evolving trends and causal dynamics over extended periods.

By acknowledging these considerations, we aim to provide clarity on the study's scope and to pave the way for complementary research endeavors that can build upon this foundation.

### 5.5 Future research suggestions

Building on the findings and limitations of the present study, several avenues for future research emerge:

Diverse demographics: While this study centered on Generation Z students, future research could diversify the sample to include working professionals, entrepreneurs, and other segments within Generation Z to gain a holistic understanding.

Generational comparative studies: In light of the study's focus on Generation Z, future research should aim to include a broader range of generational cohorts. Comparative studies involving multiple generations would provide valuable insights into generational differences and similarities in AI interactions, brand trust, and purchasing behaviors. Such studies could help in understanding the broader implications of AI across the consumer spectrum and aid in developing more comprehensive marketing strategies.

Longitudinal design: Adopting a longitudinal approach would provide insights into the evolving nature of AI interactions and its impact on brand trust and purchasing decisions over time.

Broader geographical scope: Expanding the research to different regions or countries could offer cross-cultural insights into how different Generation Z cohorts perceive and interact with AI in the context of brand trust.

In conclusion, as AI continues to weave itself into the fabric of consumer interactions, understanding its multifaceted impact on brand trust and purchasing decisions, especially for the digital natives of Generation Z, remains paramount. This study serves as a foundational step in that direction, paving the way for more nuanced and expansive explorations in the future.

## Data availability statement

The raw data supporting the conclusions of this article will be made available by the authors, without undue reservation.

## Ethics statement

Ethical approval was not required for the studies involving humans because in effect the category of the study lies on education research involving anonymous surveys on adult students. Therefore, according to IRB guidelines and the guidelines of Universidad de Monterrey these types of studies and research are exempt from IRB approval. The studies were conducted in accordance with the local legislation and institutional requirements. The participants provided their written informed consent to participate in this study.

## Author contributions

CG-T: Conceptualization, Data curation, Formal analysis, Funding acquisition, Investigation, Methodology, Project administration, Resources, Software, Supervision, Validation, Visualization, Writing – original draft, Writing – review & editing. KK: Conceptualization, Investigation, Writing – review & editing. GS-M: Investigation, Writing – review & editing. DC: Investigation, Writing – review & editing. JI: Investigation, Writing – review & editing.

## References

[B1] Abbass H. A. (2019). Social integration of artificial intelligence: functions, automation allocation logic and human-autonomy trust. Cognit. Comput. 11, 159–171. 10.1007/s12559-018-9619-0

[B2] Abrardi L. Cambini C. Rondi L. (2022). Artificial intelligence, firms and consumer behavior: a survey. J. Econ. Surv. 36, 969–991. 10.1111/joes.12455

[B3] Ahn J. Kim J. Sung Y. (2022). The effect of gender stereotypes on artificial intelligence recommendations. J. Bus. Res. 141, 50–59. 10.1016/j.jbusres.2021.12.007

[B4] Alboqami H. (2023). Trust me, I'm an influencer!-Causal recipes for customer trust in artificial intelligence influencers in the retail industry. J. Retail. Consum. Serv. 72, 103242. 10.1016/j.jretconser.2022.103242

[B5] Allal-Chérif O. Simón-Moya V. Ballester A. C. C. (2021). Intelligent purchasing: how artificial intelligence can redefine the purchasing function. J. Bus. Res. 124, 69–76. 10.1016/j.jbusres.2020.11.050

[B6] Ameen N. Hosany S. Taheri B. (2023). Generation Z's psychology and new-age technologies: Implications for future research. Psychol. Mark 40, 2029–2040. 10.1002/mar.21868

[B7] Ameen N. Sharma G. D. Tarba S. Rao A. Chopra R. (2022). Toward advancing theory on creativity in marketing and artificial intelligence. Psychol. Mark 39, 1802–1825. 10.1002/mar.21699

[B8] Ameen N. Tarhini A. Reppel A. Anand A. (2021). Customer experiences in the age of artificial intelligence. Comput. Hum. Behav. 114, 106548. 10.1016/j.chb.2020.106548 32905175 PMC7463275

[B9] Arruda Filho E. J. M. Simões J. D. S. Muylder C. F. (2020). The low effect of perceived risk in the relation between hedonic values and purchase intention. J. Market. Manage. 36, 128–148. 10.1080/0267257X.2019.1697725

[B10] Ashfaq M. Yun J. Yu S. Loureiro S. M. C. (2020). Chatbot: Modeling the determinants of users' satisfaction and continuance intention of AI-powered service agents. Telematics Inf. 54, 101473. 10.1016/j.tele.2020.101473

[B11] Baabdullah A. M. Alalwan A. A. Algharabat R. S. Metri B. Rana N. P. (2022). Virtual agents and flow experience: An empirical examination of AI-powered chatbots. Technol. Forecast Soc. Change 181, 121772. 10.1016/j.techfore.2022.121772

[B12] Bertoncini A. L. C. Serafim M. C. (2023). Ethical content in artificial intelligence systems: A demand explained in three critical points. Front. Psychol. 14, 1074787. 10.3389/fpsyg.2023.1074787 37063544 PMC10097940

[B13] Bhandari M. Rodgers S. (2020). What does the brand say? Effects of brand feedback to negative eWOM on brand trust and purchase intentions. Int. J. Advert. 37, 125–141. 10.1080/02650487.2017.1349030

[B14] Bhushan S. (2021). The impact of artificial intelligence and machine learning on the global economy and its implications for the hospitality sector in India. Worldwide Hosp. Tourism Themes 13, 252–259. 10.1108/WHATT-09-2020-0116

[B15] Bilgihan A. (2016). Gen Y customer loyalty in online shopping: an integrated model of trust, user experience and branding. Comput. Hum. Behav. 61, 103–113. 10.1016/j.chb.2016.03.014

[B16] Bilgihan A. Okumus F. Nusair K. Bujisic M. (2014). Online experiences: flow theory, measuring online customer experience in e-commerce and managerial implications for the lodging industry. Inf. Technol. Tour. 14, 49–71. 10.1007/s40558-013-0003-3

[B17] Cabiddu F. Moi L. Patriotta G. Allen D. G. (2022). Why do users trust algorithms? A review and conceptualization of initial trust and trust over time. Eur. Manage. J. 40, 685–706. 10.1016/j.emj.2022.06.001

[B18] Chakraborty U. (2019). The impact of source credible online reviews on purchase intention: the mediating roles of brand equity dimensions. J. Res. Int. Marketing 13, 142–161. 10.1108/JRIM-06-2018-0080

[B19] Cheng Y. Jiang H. (2020). How do AI-driven chatbots impact user experience? Examining gratifications, perceived privacy risk, satisfaction, loyalty, and continued use. J. Broadcast. Electron Media 64, 592–614. 10.1080/08838151.2020.1834296

[B20] Cheng Y. Jiang H. (2022). Customer–brand relationship in the era of artificial intelligence: understanding the role of chatbot marketing efforts. J. Prod. Brand Manage. 31, 252–264. 10.1108/JPBM-05-2020-2907

[B21] Chi O. H. Jia S. Li Y. Gursoy D. (2021). Developing a formative scale to measure consumers' trust toward interaction with artificially intelligent (AI) social robots in service delivery. Comput. Hum. Behav. 118, 106700. 10.1016/j.chb.2021.106700

[B22] Csikszentmihalyi M. Csikszentmihalyi M. (2014). T Toward a psychology of optimal experience. Flow Found. Positive Psychol. 2014, 209–226. 10.1007/978-94-017-9088-8_14

[B23] Dabholkar P. A. Sheng X. (2012). Consumer participation in using online recommendation agents: effects on satisfaction, trust, and purchase intentions. The Serv. Ind. J. 32, 1433–1449. 10.1080/02642069.2011.624596

[B24] Diallo M. F. Moulins J. L. Roux E. (2021). Unpacking brand loyalty in retailing: a three-dimensional approach to customer–brand relationships. Int. J. Retail Distrib. Manage. 49, 204–222. 10.1108/IJRDM-03-2020-0115

[B25] Fedorko R. Král Š. Bačík R. (2022). Artificial intelligence in e-commerce: a literature review. Artif. Intell 21, 2023. 10.1007/978-981-16-9113-3_50

[B26] Figueroa-Armijos M. Clark B. B. da Motta Veiga S. P. (2023). Ethical perceptions of AI in hiring and organizational trust: the role of performance expectancy and social influence. J. Bus. Ethics. 2022, 1–19. 10.1007/s10551-022-05166-2

[B27] Folse J. A. G. Burton S. Netemeyer R. G. (2013). Defending brands: Effects of alignment of spokescharacter personality traits and corporate transgressions on brand trust and attitudes. J. Advert. 42, 331–342. 10.1080/00913367.2013.795124

[B28] Fonseka K. Jaharadak A. A. Raman M. (2022). Impact of E-commerce adoption on business performance of SMEs in Sri Lanka; moderating role of artificial intelligence. Int. J. Soc. Econ. 49, 1518–1531. 10.1108/IJSE-12-2021-0752

[B29] Gretry A. Horváth C. Belei N. van Riel A. C. R. (2017). ‘Don't pretend to be my friend!' When an informal brand communication style backfires on social media. J. Bus. Res. 74, 77–89. 10.1016/j.jbusres.2017.01.012

[B30] Guerra-Tamez C. R. Dávila-Aguirre M. C. Barragán Codina J. N. Guerra Rodríguez P. (2020). Analysis of the elements of the theory of flow and perceived value and their influence in craft beer consumer loyalty. J. Int. Food Agribus. Marketing 2020, 1–31. 10.1080/08974438.2020.1823929

[B31] Guerra-Tamez C. R. Franco-García M. L. (2022). Influence of flow experience, perceived value and csr in craft beer consumer loyalty: a comparison between Mexico and the Netherlands. Sustainability 14, 8202. 10.3390/su14138202

[B32] Guo W. Luo Q. (2023). Investigating the impact of intelligent personal assistants on the purchase intentions of Generation Z consumers: The moderating role of brand credibility. J. Retail. Consum. Serv. 73, 103353. 10.1016/j.jretconser.2023.103353

[B33] Hasan R. Shams R. Rahman M. (2021). Consumer trust and perceived risk for voice-controlled artificial intelligence: the case of Siri. J. Bus. Res. 131, 591–597. 10.1016/j.jbusres.2020.12.012

[B34] Hayes J. L. Britt B. C. Evans W. Rush S. W. Towery N. A. Adamson A. C.. (2021). Can social media listening platforms' artificial intelligence be trusted? Examining the accuracy of Crimson Hexagon's (now Brandwatch Consumer Research's) AI-Driven analyses. J. Advert. 50, 81–91. 10.1080/00913367.2020.1809576

[B35] Ho S. P. S. Chow M. Y. C. (2023). The role of artificial intelligence in consumers' brand preference for retail banks in Hong Kong. J. Financ. Serv. Market. 12, 1–14. 10.1057/s41264-022-00207-3

[B36] Iglesias O. Markovic S. Singh J. J. Sierra V. (2019). Do customer perceptions of corporate services brand ethicality improve brand equity? Considering the roles of brand heritage, brand image, and recognition benefits. J. Bus. Ethics 154, 441–459. 10.1007/s10551-017-3455-0

[B37] Ismail A. R. Nguyen B. Chen J. Melewar T. C. Mohamad B. (2021). Brand engagement in self-concept (BESC), value consciousness and brand loyalty: a study of generation Z consumers in Malaysia. Young Consum. 22, 112–130. 10.1108/YC-07-2019-1017

[B38] Joshi R. Garg P. (2021). Role of brand experience in shaping brand love. Int. J. Consum. Stud. 45, 259–272. 10.1111/ijcs.12618

[B39] Kautish P. Khare A. (2022). Investigating the moderating role of AI-enabled services on flow and awe experience. Int. J. Inf. Manage. 66, 102519. 10.1016/j.ijinfomgt.2022.102519

[B40] Khaliq A. Waqas A. Nisar Q. A. Haider S. Asghar Z. (2022). Application of AI and robotics in hospitality sector: a resource gain and resource loss perspective. Technol. Soc. 68, 101807. 10.1016/j.techsoc.2021.101807

[B41] Khatri P. (2023). “The gaming experience with AI,” in Research Anthology on Game Design, Development, Usage, and Social Impact. London: IGI Global, 14–30.

[B42] Khrais L. T. (2020). Role of artificial intelligence in shaping consumer demand in E-commerce. Future Int. 12, 226. 10.3390/fi12120226

[B43] Kim J. Giroux M. Lee J. C. (2021). When do you trust AI? The effect of number presentation detail on consumer trust and acceptance of AI recommendations. Psychol. Mark 38, 1140–1155. 10.1002/mar.21498

[B44] Kim R. B. Chao Y. (2019). Effects of brand experience, brand image and brand trust on brand building process: The case of Chinese millennial generation consumers. J. Int. Stu. 12, 2019. 10.14254/2071-8330.2019/12-3/1

[B45] Kim-Vick J. Yu U. J. (2023). Impact of digital resale platforms on brand new or second-hand luxury goods purchase intentions among US Gen Z consumers. Int. J. Fashion Design Technol. Educ. 16, 57–69. 10.1080/17543266.2022.2113154

[B46] Kumar V. Rajan B. Venkatesan R. Lecinski J. (2019). Understanding the role of artificial intelligence in personalized engagement marketing. Calif. Manage. Rev. 61, 135–155. 10.1177/0008125619859317

[B47] Mantello P. Ho M. T. Nguyen M. H. Vuong Q. H. (2023). Bosses without a heart: socio-demographic and cross-cultural determinants of attitude toward Emotional AI in the workplace. AI Soc. 38, 97–119. 10.1007/s00146-021-01290-1 34776651 PMC8571983

[B48] Marjerison R. K. Zhang Y. Zheng H. (2022). AI in E-commerce: application of the use and gratification model to the acceptance of chatbots. Sustainability 14, 14270. 10.3390/su142114270

[B49] Mayer A. S. Strich F. Fiedler M. (2020). Unintended consequences of introducing AI systems for decision making. MIS Q. Executive 19, 2020. 10.17705/2msqe.00036

[B50] McLean G. Osei-Frimpong K. Barhorst J. (2021). Alexa, do voice assistants influence consumer brand engagement?–Examining the role of AI powered voice assistants in influencing consumer brand engagement. J. Bus. Res. 124, 312–328. 10.1016/j.jbusres.2020.11.045

[B51] Menidjel C. Benhabib A. Bilgihan A. (2017). Examining the moderating role of personality traits in the relationship between brand trust and brand loyalty. J. Product Brand Manage. 26, 631–649. 10.1108/JPBM-05-2016-1163

[B52] Meyer-Waarden L. Cloarec J. (2021). ‘Baby, you can drive my car': Psychological antecedents that drive consumers' adoption of AI-powered autonomous vehicles. Technovation 109, 102348. 10.1016/j.technovation.2021.102348

[B53] Micu A. Micu A. E. Geru M. Căpăṭînă A. Muntean M. C. (2021). The impact of artificial intelligence use on the e-commerce in Romania. Amfiteatru Econ. 23, 137–154. 10.24818/EA/2021/56/137

[B54] Minton E. A. Kaplan B. Cabano F. G. (2022). The influence of religiosity on consumers' evaluations of brands using artificial intelligence. Psychol. Mark 39, 2055–2071. 10.1002/mar.21727

[B55] Nadarzynski T. Miles O. Cowie A. Ridge D. (2019). acceptability of artificial intelligence (AI)-led chatbot services in healthcare: a mixed-methods study. Digit. Health 5, 2055207619871808. 10.1177/2055207619871808 31467682 PMC6704417

[B56] Nagy S. Hajd,ú N. (2021). Consumer acceptance of the use of artificial intelligence in online shopping: Evidence from Hungary. Amfiteatru Econ. 23, 155–173. 10.24818/EA/2021/56/155

[B57] Nazir S. Khadim S. Asadullah M. A. Syed N. (2023). Exploring the influence of artificial intelligence technology on consumer repurchase intention: The mediation and moderation approach. Technol. Soc. 72, 102190. 10.1016/j.techsoc.2022.102190

[B58] Nguyen T. Quach S. Thaichon P. (2022). The effect of AI quality on customer experience and brand relationship. J. Consum. Behav. 21, 481–493. 10.1002/cb.1974

[B59] Nitzl C. Roldan J. L. Cepeda G. (2016). Mediation analysis in partial least squares path modeling. Ind. Manage. Data Syst. 116, 1849–1864. 10.1108/IMDS-07-2015-0302

[B60] Nosi C. Pucci T. Melanthiou Y. Zanni L. (2021). The influence of online and offline brand trust on consumer buying intention. EuroMed J. Bus. 17, 550–567. 10.1108/EMJB-01-2021-0002

[B61] Ozkara B. Y. Ozmen M. Kim J. W. (2017). Examining the effect of flow experience on online purchase: a novel approach to the flow theory based on hedonic and utilitarian value. J. Retailing Consum. Serv. 37, 119–131. 10.1016/j.jretconser.2017.04.001

[B62] Păvăloaia V. D. Necula S. C. (2023). Artificial intelligence as a disruptive technology—a systematic literature review. Electronics 12, 1102. 10.3390/electronics12051102

[B63] Pelau C. Dabija D. C. Ene I. (2021). What makes an AI device human-like? The role of interaction quality, empathy and perceived psychological anthropomorphic characteristics in the acceptance of artificial intelligence in the service industry. Comput. Hum. Behav. 122, 106855. 10.1016/j.chb.2021.106855

[B64] Pitardi V. Marriott H. R. (2021). Alexa, she's not human but… Unveiling the drivers of consumers' trust in voice-based artificial intelligence. Psychol. Mark 38, 626–642. 10.1002/mar.21457

[B65] Portal S. Abratt R. Bendixen M. (2019). The role of brand authenticity in developing brand trust. J. Strat. Marketing 27, 714–729. 10.1080/0965254X.2018.1466828

[B66] Pradhan D. Kuanr A. Anupurba Pahi S. Akram M. S. (2023). Influencer marketing: When and why gen Z consumers avoid influencers and endorsed brands. Psychol. Mark 40, 27–47. 10.1002/mar.21749

[B67] Prentice C. Dominique Lopes S. Wang X. (2020). The impact of artificial intelligence and employee service quality on customer satisfaction and loyalty. J. Hosp. Market. Manage. 29, 739–756. 10.1080/19368623.2020.1722304

[B68] Priporas C.-. V Stylos N. Fotiadis A. K. (2017). Generation Z consumers' expectations of interactions in smart retailing: a future agenda. Comput. Hum. Behav. 77, 374–381. 10.1016/j.chb.2017.01.058

[B69] Puiu S. Demyen S. Tănase A. C. Vărzaru A. A. Bocean C. G. (2021). Assessing the adoption of mobile technology for commerce by generation Z. Electronics 11, 866. 10.3390/electronics11060866

[B70] Qalati S. A. Vela E. G. Li W. Dakhan S. A. Hong Thuy T. T. Merani S. H. (2021). Effects of perceived service quality, website quality, and reputation on purchase intention: The mediating and moderating roles of trust and perceived risk in online shopping. Cogent Bus. Manage. 8, 1869363. 10.1080/23311975.2020.1869363

[B71] Qin F. Li K. Yan J. (2020). Understanding user trust in artificial intelligence-based educational systems: evidence from China. Br. J. Educ. Technol. 51, 1693–1710. 10.1111/bjet.12994

[B72] Quaye E. S. Taoana C. Abratt R. Anabila P. (2022). Customer advocacy and brand loyalty: the mediating roles of brand relationship quality and trust. J. Brand Manage. 29, 363–382. 10.1057/s41262-022-00276-8

[B73] Rana J. Gaur L. Singh G. Awan U. Rasheed M. I. (2021). Reinforcing customer journey through artificial intelligence: a review and research agenda. Int. J. Emerg. Markets 17, 1738–1758. 10.1108/IJOEM-08-2021-1214

[B74] Rasheed H. M. W. He Y. Khizar H. M. U. Abbas H. S. M. (2023). Exploring Consumer-Robot interaction in the hospitality sector: unpacking the reasons for adoption (or resistance) to artificial intelligence. Technol. Forecast Soc. Change 192, 122555. 10.1016/j.techfore.2023.122555

[B75] Rashidin M. S. Gang D. Javed S. Hasan M. (2021). The role of artificial intelligence in sustaining the e-commerce ecosystem: Alibaba vs. Tencent. J. Glob. Inf. Manage. 30, 1–25. 10.4018/JGIM.304067

[B76] Rodgers W. Yeung F. Odindo C. Degbey W. Y. (2021). Artificial intelligence-driven music biometrics influencing customers' retail buying behavior. J. Bus. Res. 126, 401–414. 10.1016/j.jbusres.2020.12.039

[B77] Sampat B. Behl A. Raj S. (2023). Understanding fitness app users' loyalty and word of mouth through gameful experience and flow theory. AIS Trans. Hum. Comput. Int. 15, 193–223. 10.17705/1thci.00088

[B78] Serravalle F. Vannucci V. Pantano E. (2022). “Take it or leave it?': Evidence on cultural differences affecting return behaviour for Gen Z. J. Retail. Consum. Serv. 66, 102942. 10.1016/j.jretconser.2022.102942

[B79] Shi S. Gong Y. Gursoy D. (2021). Antecedents of trust and adoption intention toward artificially intelligent recommendation systems in travel planning: a heuristic–systematic model. J. Travel Res. 60, 1714–1734. 10.1177/0047287520966395

[B80] Shim S. I. Forsythe S. Kwon W. S. (2015). Impact of online flow on brand experience and loyalty. J. Electr. Comm. Res. 16, 56.

[B81] Shin D. (2021). The effects of explainability and causability on perception, trust, and acceptance: Implications for explainable AI. Int. J. Hum. Comput. Stud. 146, 102551. 10.1016/j.ijhcs.2020.102551

[B82] Singh B. (2021). Predicting airline passengers' loyalty using artificial neural network theory. J. Air Trans. Manage. 94, 102080. 10.1016/j.jairtraman.2021.102080

[B83] Stephanidis C. (2019). Seven HCI grand challenges. Int. J. Hum. Comput. Int. 35, 1229–1269. 10.1080/10447318.2019.1619259

[B84] Strich F. Mayer A. S. Fiedler M. (2021). What do I do in a world of artificial intelligence? Investigating the impact of substitutive decision-making AI systems on employees' professional role identity. J. Assoc. Inf. Syst. 22, 9. 10.17705/1jais.00663

[B85] Tabassum S. Khwaja M. G. Zaman U. (2020). Can narrative advertisement and eWOM influence generation Z purchase intentions? Information 11, 545. 10.3390/info11120545

[B86] Tenenhaus M. Amato S. Esposito Vinzi V. (2004). A global goodness-of-fit index for PLS structural equation modelling. Proc. XLII SIS Sci. Meeting 1, 739–742.

[B87] Tenenhaus M. Vinzi V. E. Chatelin Y. M. Lauro C. (2005). PLS path modeling. Comput. Stat. Data Anal. 48, 159–205. 10.1016/j.csda.2004.03.005

[B88] Tussyadiah I. Miller G. (2019). “Perceived impacts of artificial intelligence and responses to positive behaviour change intervention,” in Information and Communication Technologies in Tourism 2019: Proceedings of the International Conference. Cham: Springer, 359–370.

[B89] Varsha P. S. Akter S. Kumar A. Gochhait S. Patagundi B. (2021). The impact of artificial intelligence on branding: a bibliometric analysis (1982-2019). JGIM 29, 221–246. 10.4018/JGIM.20210701.oa10

[B90] Verma D. Tripathi V. Singh A. P. (2021). From physical to digital: what drives generation Z for mobile commerce adoption? J. Asia Bus. Stu. 15, 732–747. 10.1108/JABS-05-2020-0207

[B91] Vlačić B. Corbo L. Silva S. C. Dabić M. (2021). The evolving role of artificial intelligence in marketing: a review and research agenda. J. Bus. Res. 128, 187–203. 10.1016/j.jbusres.2021.01.055

[B92] Wang Z. Li M. Lu J. Cheng X. (2022). Business innovation based on artificial intelligence and blockchain technology. Inf. Proc. Manage. 59, 102759. 10.1016/j.ipm.2021.102759

[B93] Wetzels M. Odekerken-Schröder G. Van Oppen C. (2009). Using PLS path modeling for assessing hierarchical construct models: guidelines and empirical illustration. MIS Q. 2009, 177–195. 10.2307/20650284

[B94] Xia Y. (2023). Impact of AI-assisted music classification in video games for sustaining effectiveness. Soft Comput. 2023, 1–16. 10.1007/s00500-023-08093-0

[B95] Yang R. Wibowo S. (2022). User trust in artificial intelligence: a comprehensive conceptual framework. Electr. Markets 32, 2053–2077. 10.1007/s12525-022-00592-6

[B96] Yang T. Nazir S. (2022). A comprehensive overview of AI-enabled music classification and its influence in games. Soft Comput. 26, 7679–7693. 10.1007/s00500-022-06734-4

[B97] Yeo S. F. Tan C. L. Kumar A. Tan K. H. Wong J. K. (2022). Investigating the impact of AI-powered technologies on Instagrammers' purchase decisions in digitalization era–A study of the fashion and apparel industry. Technol. Forecast. Soc. Change 177, 121551. 10.1016/j.techfore.2022.121551

[B98] Youn S. Jin S. V. (2021). In AI we trust?” The effects of parasocial interaction and technopian versus luddite ideological views on chatbot-based customer relationship management in the emerging “feeling economy. Comput. Hum. Behav. 119, 106721. 10.1016/j.chb.2021.106721

[B99] Zhao J.-. D Huang J. S. Su S. (2019). The effects of trust on consumers' continuous purchase intentions in C2C social commerce: a trust transfer perspective. J. Retail. Consum. Serv. 50, 42–49. 10.1016/j.jretconser.2019.04.014

[B100] Zuech R. Khoshgoftaar T. M. Wald R. (2015). Intrusion detection and big heterogeneous data: a survey. J. Big Data 2, 1–41. 10.1186/s40537-015-0013-4

